# The Morpho-Molecular Landscape of Spitz Neoplasms

**DOI:** 10.3390/ijms23084211

**Published:** 2022-04-11

**Authors:** Carlo Alberto Dal Pozzo, Rocco Cappellesso

**Affiliations:** 1 Surgical Pathology and Cytopathology Unit, Department of Medicine (DIMED), University of Padua, 35121 Padua, Italy; carloalberto.dalpozzo@studenti.unipd.it; 2 Pathological Anatomy Unit, University Hospital of Padua, 35121 Padua, Italy

**Keywords:** Spitz nevus, atypical Spitz tumor, malignant Spitz tumor, HRAS, MAP2K1, ALK, ROS1, RET, MET, MAP3K8, NTRK1, NTRK2, NTRK3

## Abstract

Spitz neoplasms are a heterogeneous group of melanocytic proliferations with a great variability in the histological characteristics and in the biological behavior. Thanks to recent discoveries, the morpho-molecular landscape of Spitz lineage is becoming clearer, with the identification of subtypes with recurrent features thus providing the basis for a more solid and precise tumor classification. Indeed, specific mutually exclusive driver molecular events, namely *HRAS* or *MAP2K1* mutations, copy number gains of 11p, and fusions involving *ALK, ROS, NTRK1, NTRK2, NTRK3, MET, RET, MAP3K8,* and *BRAF* genes, correlate with distinctive histological features. The accumulation of further molecular aberrations, instead, promotes the increasing malignant transformation of Spitz neoplasms. Thus, the detection of a driver genetic alteration can be achieved using the appropriate diagnostic tests chosen according to the histological characteristics of the lesion. This allows the recognition of subtypes with aggressive behavior requiring further molecular investigations. This review provides an update on the morpho-molecular correlations in Spitz neoplasms.

## 1. Introduction

**Figure 1:**
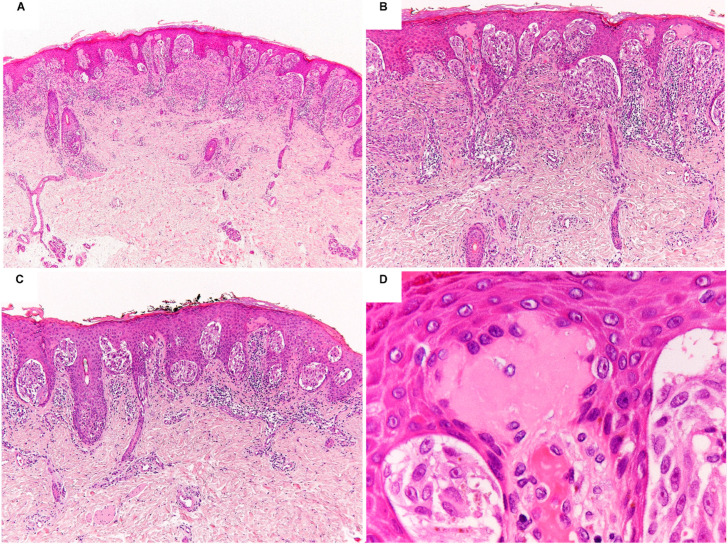
Photomicrographs of a prototypical Spitz nevus showing a symmetric, slightly raised, compound, and maturating melanocytic proliferation with epidermal hyperplasia, flat base, and scattered Kamino bodies, composed of large clefting nests of spindle and epithelioid melanocytes with abundant eosinophilic cytoplasm, moderately pleomorphic vesicular nuclei, and prominent nucleoli (**A**–**D**) H&E staining; original magnification 10×, 100×, 100×, and 400×, respectively).

In 1948, the American pathologist Sophie Spitz published a landmark case series of melanocytic proliferations called “juvenile melanomas” or “melanomas of childhood” characterized by a combination of distinctive architectural and cytological features in association with peculiar epidermal changes (Figure 1) [1,2]. Such lesions are symmetric, often showing a dome-shaped, wedge-shaped or plaque-like silhouette, with sharp lateral borders and maturation towards the deep part [1,2]. The pattern of growth is predominantly nested. Nests may display varying cellularity, size, and shape are arranged in parallel to the rete ridges [1,2]. The overlying epidermis is hyperplastic and may be separated by the junctional nests and single melanocytes by clefts [1,2]. Pagetoid spread of the melanocytes into the epidermis, when present, usually occurs in bundles or nests of cells [1,2]. A variable number of small to large dull eosinophilic globules composed of amorphous filaments (so-called Kamino bodies) may be scattered throughout [1,2]. There is a predominance of enlarged epithelioid and/or spindle melanocytes with abundant eosinophilic or amphophilic cytoplasm with “ground-glass” appearance, round to oval to spindle nuclei with finely dispersed chromatin and distinct nucleoli [1,2]. Nuclear pseudoinclusions and multinucleated melanocytes may be encountered. Most lesions are amelanotic or paucimelanotic [1,2]. The mitotic activity is usually low. The inflammatory cell infiltrate is generally perivascular and dispersed throughout. These melanocytic proliferations typically occurred on the extremities of children and young adults and, despite the tendency to loco-regional nodal involvement, were hallmarked by an indolent clinical course, thus justifying their distinction from the adult melanoma in which the prognosis was dismal [1,3]. With time, it became evident that using these histological criteria it was possible to distinguish a group of benign melanocytic lesions – then named Spitz nevus (SN)–from melanoma and that these may be present also in adults [4].

However, cases occur that are more difficult to differentiate from melanoma because showing SN characteristics along with worrisome histological features, such as increased size, asymmetry, epidermal ulceration, lack of maturation, solid growth, diffuse pagetoid spread, hypodermic extension, marked cytological atypia, increased mitotic activity, deep and atypical mitoses. In 1959 Albert Bernard Ackerman recognized that melanocytic proliferations with Spitz histology encompassed a broad morphological spectrum of neoplasms ranging from completely benign, namely SN, to their fully malignant counterpart, namely malignant Spitz tumor (MST), passing through an intermediate category, namely atypical Spitz tumor (AST; Table 1) [5,6,7]. It must be highlighted that many MST diagnoses are achieved in the context of known synchronous metastasis or are initially defined as AST and classified as fully malignant after the detection of distant metastasis during the clinical follow-up. Indeed, it is well known that distinguishing AST from MST histologically is very difficult and at times impossible, even with the aid of common ancillary analyses. Spitz neoplasms may present at any age and at any site but most frequently affect the lower extremities and the face of patients under the age of 30 [8]. They are quite infrequent, accounting for about 1% of all resected melanocytic lesions and with an estimated annual incidence of little more than 1 case per 100,000 [8,9]. AST do not exceed the 6–8% of the number of SN and MST are very rare [10]. In the footsteps of Ackermann, the term “Spitzoid” was coined referred to melanocytic neoplasms sharing (at least some of) the distinctive SN features. This has led to confusion in the classification of melanocytic lesions since the term Spitzoid has been applied even to lesions with only the epidermal modifications typical of SN or to those with a small subpopulation of enlarged epithelioid and/or spindle melanocytes.

Until a few years ago, the genetic determinants of Spitz neoplasms development were largely unknown [11,12,13]. Thanks to the recent discoveries on the molecular landscape of the melanocytic Spitz lineage, the taxonomy of these tumors and the understanding of their biological behavior is becoming clearer [14]. Overall, both nevi and melanomas share the activation of some growth-promoting signaling pathways (considered the driver molecular events), mainly PI3K-AKT and RAF-MEK1/2-ERK1/2 [15,16]. In benign nevi these are almost the only molecular aberrations present [15,16]. In malignant melanomas, instead, there is also a variable number of additional molecular alterations (considered the promoting molecular events) able to block tumor-suppression mechanisms and to trigger further oncogenic signals, such as 9p21 deletion, 6p25 copy number gain, *TP53* mutations, and *TERT*-promoter mutation [15,17,18,19] Thus, the driver molecular events seem to determine the specific histotype of each melanocytic neoplasm, while the type and amount of promoting aberrations seem to define their morphological and clinical aggressiveness [15,16]. According to this interpretation, the neoplasms regarded as melanocytic tumors of uncertain malignant potential result from the combination of the same driver molecular alterations of nevi and melanomas with a limited number of additional promoting genetic events determining a malignant potential lower than that of full-blown melanoma [15,16]. This seems to be true also in the Spitz setting. Indeed, the drivers of most common nevi and malignant melanomas are *BRAF* and *NRAS* activating mutations, but these alterations are virtually absent in Spitz neoplasms [20,21,22,23,24]. Instead, these harbor oncogenic *HRAS* or *MAP2K1* mutations or kinase gene fusions involving *ALK, BRAF, MET, NTRK1, NTRK2, NTRK3, RET, ROS1,* and *MAP3K8* in a mutually exclusive pattern [5,11,12,13,25,26,27,28,29,30,31,32,33,34,35,36,37]. The integration of such molecular data in the histology-based tumor classification of melanocytic neoplasms provides a way to identify the true Spitz neoplasms in the heterogeneous group of Spitzoid lesions [14]. Moreover, the existence of consistent genotype-phenotype relationships among the different subtypes of Spitz neoplasms (Table 2) can be used for the selection of the appropriate ancillary analyses to support a histological diagnosis of AST or MST and to better assess their malignant risk.

This review resumes the state-of-art in the knowledge of genotype-phenotype correlations in the field of Spitz neoplasms, especially focusing on subtypes harboring *HRAS* or *MAP2K1* mutations, copy number gains of 11p, or fusions involving *ALK, ROS, NTRK1, NTRK2, NTRK3, MET, RET, MAP3K8,* and *BRAF* genes.

## 2. Spitz Neoplasms with *HRAS* Mutations or 11p Copy Number Gains

The *RAS* proto-oncogene family comprises three members, namely *KRAS*, *NRAS*, and *HRAS*, respectively located on the short arm (p) of chromosome 12, 1, and 11, and encoding the proteins KRAS4A, KRAS4B, NRAS, and HRAS [38,39]. These proteins are implicated in the signal transduction from the cell surface to the nucleus through the PI3K-AKT and RAF-MEK1/2-ERK1/2 pathways stimulating growth, differentiation, proliferation, and survival of the cell [40,41]. Missense single nucleotide point mutations usually occur in hotspot regions of the RAS active site leading to the production of aberrant proteins able to trigger downstream signaling without the need of extracellular cues [39]. Interestingly, the oncogenic role of RAS seems to be histotype-specific since different tumors are related to mutation of a precise RAS isoform [40]. This is particularly evident in melanocytic lesions where common nevi and malignant melanomas harbor almost exclusively *NRAS* mutations while Spitz neoplasms *HRAS* mutations [12,22,24]. Compared to the other RAS isoforms, HRAS appears to have a higher affinity for the PI3K-AKT pathway that is believed to be responsible for conferring the enlarged epithelioid or spindle phenotype to the melanocytes in Spitz neoplasms [42,43,44]. Copy number gains of the 11p region encompassing *HRAS* leads to overexpression of the protein product with similar results. Activating *HRAS* mutations and copy number gains of 11p occur in an exclusive or concurrent way in about 20% of Spitz neoplasms [22,45,46,47,48]. Most *HRAS* mutations commonly involve the codons 59–61 in exon 3 (mainly Q61R and Q61L) and rarely affect the codons 12 and 13 in exon 2 (mainly G13R) [5,13,22,48,49,50,51,52,53,54,55,56]. The detection of *HRAS* mutation requires sequencing analysis (classic or NGS), while FISH or CGH analysis is needed for the identification of 11p copy number alterations. The monoclonal antibody SP174 recognizes with high sensitivity the RAS Q61R mutant protein; unfortunately, however, it cross-reacts with both KRAS, NRAS, and HRAS isoforms resulting useless from a diagnostic point of view [57,58] Antibodies against the wild type HRAS protein may be of aid in highlighting Spitz neoplasms with HRAS overexpression due to 11p copy number gains.

**Figure 2:**
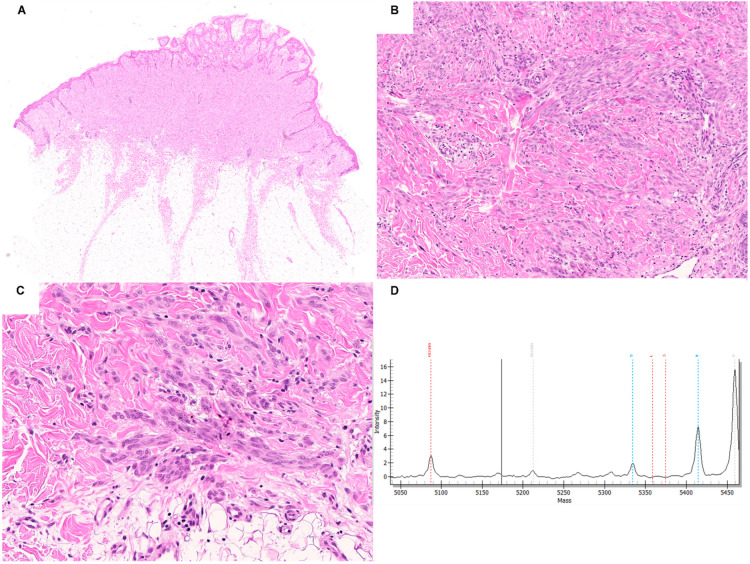
Photomicrographs of a Spitz nevus harboring HRAS mutation showing a symmetric, exophytic and dermal melanocytic proliferation with flat base, composed of fascicles of large spindle melanocytes with amphophilic cytoplasm, moderately pleomorphic vesicular nuclei, and distinct nucleoli, intermingled with thick collagen bundles (**A**–**C**) H&E staining; original magnification 10×, 100×, and 200×, respectively). Mass Array graphic output showing the HRAS p.Q61R c.182A > G mutation detected by mass spectrometry-based analysis (**D**).

Spitz neoplasms with *HRAS* aberrations tend to be predominantly intradermal symmetric lesions with infiltrative base characterized by epithelioid and spindle cells with abundant eosinophilic or amphophilic cytoplasm and slightly to moderately pleomorphic vesicular nuclei, intermingled with thick collagen bundles (desmoplasia) (Figure 2) [12,13,48]. Marked cytological atypia can be present in about 40% of *HRAS*-mutated cases [33]. Usually, mitoses are rare and not atypical. However, it must be highlighted that, on one hand, not all the Spitz neoplasms with these genomic alterations are desmoplastic and, on the other hand, desmoplasia has been reported also in Spitz neoplasms with *ROS1, ALK,* and *BRAF* gene fusions [13,59].

Most Spitz neoplasms with activating *HRAS* mutations and/or copy number gains of 11p are readily recognizable as SN, but cases occur with AST features; the prognosis is favorable [22,46,60,61].

## 3. Spitz Neoplasms with *ALK* Fusions

*ALK* resides on chromosome 2p and encodes a tyrosine kinase receptor involved in the PI3K-AKT, RAF-MEK1/2-ERK1/2, and JAK3-STAT3 pathways [62,63,64,65]. The reported proportion of cases with *ALK* fusions among SN and AST ranges from 10% to 20%, but it is restricted to approximately 1% of Spitz melanomas [66]. The *ALK* most frequent fusion partners are *TPM3* and *DCTN1* and it has been proposed that these rearrangements, compared with other fusions involved in the pathogenesis of Spitz neoplasms, lead to a very delayed oncogene-induced senescence resulting in large lesions [5,13,61,66,67,68,69,70,71,72]. Other recurrent fusion partners of *ALK* in the field of Spitz neoplasms include: *MLPH, MYO5A, CLIP1, DDX3Y, KANK1, EEF2, GTF3C2, NPM1, PPFIBP1, SPTAN1,* and *TPR* [68,69,70,73,74,75]. ALK immunohistochemistry with the monoclonal antibodies D5F3 and 5A4 serves as excellent surrogate for *ALK* fusions and is indicated in the appropriate morphological setting [11,66,70]. The expression is diffuse, strong, and granular in the cytoplasm of the melanocytes and may be present or not in the membrane or the nucleus [11,66,70]. NGS and FISH are the appropriate molecular techniques for the detection of the *ALK* fusions.

**Figure 3:**
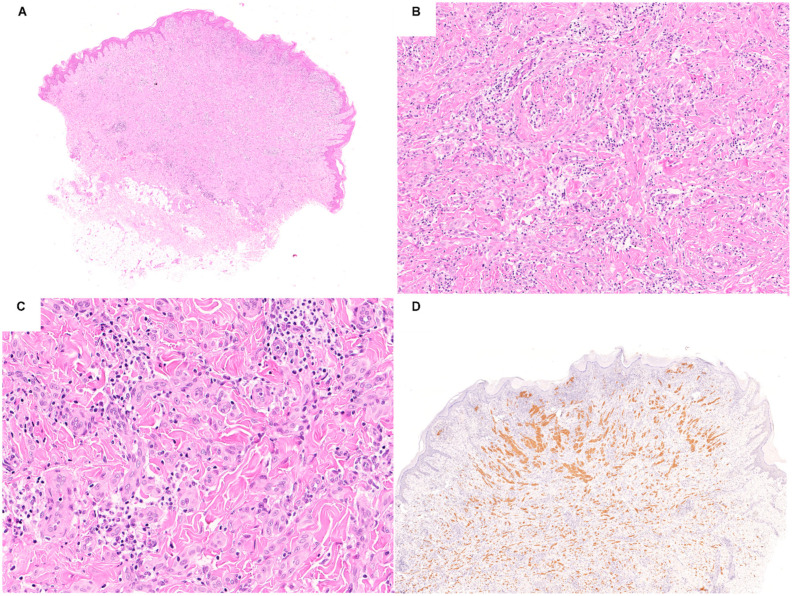
Photomicrographs of a Spitz nevus harboring ALK fusion showing a large, exophytic and dermal melanocytic proliferation with infiltrative base, composed of nonpigmented, large, epithelioid and spindle melanocytes with amphophilic cytoplasm, vesicular nuclei, and prominent nucleoli, arranged in plexiform intersecting fascicles (**A**–**C**) H&E staining; original magnification 10×, 100×, and 200×, respectively). ALK (clone D5F3) immunohistochemistry showing a diffuse cytoplasmic staining ((**D**) original magnification 12.5×).

Clinically, *ALK*-fused Spitz neoplasms tend to be large and solitary papules or nodules arising on the extremities of young patients [3,11,70]. Histologically, most of these lesions share a distinctive pattern substantiated by a compound wedge shape silhouette with a bulbous and/or infiltrative base (Figure 3) [11,71,76]. The presence of non-pigmented, large, spindle melanocytes with pericellular clefts, amphophilic cytoplasm, vesicular nuclei, and prominent nucleoli, growing in plexiform intersecting fascicles, appears as a hallmark of *ALK* fusions [11,33,71,76]. Nevertheless, it is important to note that, although the described pattern is extremely sensitive in the prediction of *ALK* fusions, it is not equally specific. Other molecular subtypes of Spitz neoplasms may show overlapping features, such as *NTRK1*-fused cases [76]. Another pattern reported to be quite characteristic for *ALK*-fused Spitz neoplasm is the angiomatoid one [33]. The epidermis is often hyperplastic and pagetoid spread is usually absent [11]. Nuclear pleomorphism is usually mild and rarely moderate [11,33,71,76]. Worrisome features, such as ulceration, deep mitoses, and perineural invasions have been described [11,68,69,70,77]. Of note, combined Spitz neoplasms are more commonly *ALK*-fused [33].

## 4. Spitz Neoplasms with *ROS1* Fusions

*ROS1* proto-oncogene is located on the long arm (q) of chromosome 6 and encodes a tyrosine kinase receptor implicated in the PI3K-AKT, RAF-MEK1/2-ERK1/2, and JAK3-STAT3 pathways [78]. According to the analysis of large series of Spitz neoplasms, ROS1 fusions were found in 7–17% of cases [13,31]. Among these lesions, several fusion partners were reported, with *PWWP2A* (37% of cases) and T*PM3* (31% of cases) being the most common in the series by Gerami et al. [59]. Other less common fusion partners of *ROS1* among Spitz neoplasms are: *PPFIBP1, CLIP1, ERC1, FIP1L1, HLA-A, MYH9, ZCCHC8, CAPRIN1, KIAA1598, MYH9,* and *MYO5A* [5,13,59,73,79]. Immunohistochemistry with monoclonal antibody D4D6 against ROS1 is a fast, low-cost, and well-performing screening test for the identification of *ROS1*-fused Spitz neoplasms. Indeed, it showed 100% sensitivity and specificity when compared with FISH [80]. Although different ROS1 immunohistochemical patterns have been observed, such as diffuse or sparse granular cytoplasmic staining, dot-like staining, and nuclear staining, no specific correlations with the cellular localization of the various *ROS1* fusions have been found [80]. In addition to FISH, NGS can be used to identify *ROS1* fusions.

Clinically, *ROS1*-fused Spitz neoplasms are pink to red papules distributed throughout the body, mainly occurring in young adults of both sexes [59]. Histologically, there are not distinctive features specifically associated with *ROS1* fusions. Indeed, many characteristics are shared with other molecular subtypes of Spitz neoplasms, particularly with those *NTRK1*-fused [59]. Nevertheless, a certain histological signature is enriched since most of the reported *ROS1*-fused lesions show a compound plaque-like or nodular silhouette with prominent expansile junctional nesting, with possible adnexal involvement and transepidermal elimination, composed of pure spindle melanocytes or of mixed spindle and epithelioid melanocytes, with mild to moderate nuclear pleomorphism, evidence of maturation, lack of pigmentation, associated with numerous Kamino bodies [13,33,59,79]. Recently, plexiform and angiomatoid patterns have been reported as more common in *ROS1*-fused Spitz neoplasms than in other molecular subtypes [33]. Cell sizes range from intermediate to large [13,59]. Mitoses may be not uncommon [79]. As above mentioned, cases occur in which *ROS1* fusions have been found in desmoplastic SN [59].

Of great interest, in all published series, the presence of *ROS1* fusions was associated with favorable outcome (no recurrence, no distant metastasis, and negative sentinel lymph node biopsy), although having been identified in cases diagnosed as MST [13]. No adverse events were observed in the available follow ups; thus, it is entirely reasonable to admit that *ROS1*-fused Spitz neoplasms usually have an indolent course [59].

## 5. Spitz Neoplasms with *NTRK* Fusions

The *NTRK* proto-oncogene family comprises three members, namely *NTRK1*, *NTRK2*, and *NTRK3*, respectively located on chromosomes 1q, 9q, and 15q and encoding the cell surface receptor tyrosine kinase proteins TRKA, TRKB, and TRKC (collectively referred as TRK proteins) involved in the PI3K-AKT, RAF-MEK1/2-ERK1/2, and PLCγ1 pathways [81,82,83]. These receptors are normally expressed in the nervous system and can be activated by the binding with several ligands, such as NGF, BDNF, and NT-3/4 [81]. TRK activation determines the autophosphorylation of the intracellular tyrosine residues and consequently the transmission of the signal through different pathways regulating the transcription of genes involved in neuronal survival and differentiation [81]. Fusions involving the *NTRK* gene family results in the production of TRK chimeric proteins with oncogenic properties since they couple constitutive expression with ligand-independent activation provided by the kinase domain preservation [83].

Among Spitz neoplasms, both *NTRK1*, *NTRK2,* and *NTRK3* fusions have been reported, but NTRK1 alterations are by far the most prevalent [5,11,13,27,28,29,30,33,68,73,76,83,84,85,86,87,88].The partners of *NTRK1* so far identified are *LMNA, TPM3*, *TP53,* and *KHDRBS1* [13,28,73,88]. Those of *NTRK3* are *ETV6, MYO5A, MYH9*, and *SQSTM1* [27,29,73,83,85,88]. The only *NTRK2* fusion identified in Spitz neoplasms has *TFG* as partner [30]. The frequency of *NTRK* fusions in Spitz neoplasms is approximately 10% [88].

Current guidelines for *NTRK* fusion detection in solid tumors state that a two-step testing approach must be followed [89]. Firstly, cases must be immunohistochemically screened using the monoclonal antibody EPR17341 that reacts against a C-terminal epitope conserved in wild-type and in all the chimeric TRK proteins (hence the definition of pan-TRK immunohistochemistry) [89,90,91]. Secondly, all the immunohistochemically positive cases must be analyzed using RNA-based NGS to prove the *NTRK* fusion [89]. This algorithm has been tested on a large AST series confirming the reliability of pan-TRK immunohistochemistry as screening test [88]. However, the application of RNA-based NGS to verify the presence of the gene fusion showed some weakness and it has been suggested to also perform FISH in all pan-TRK positive cases not confirmed by NGS analysis [88]. The staining pattern of pan-TRK can also provide information about the probable underlying *NTRK* fusion. Indeed, strong and diffuse nuclear immunostaining is quite specific for *ETV6-NTRK3* fusion, while linear immunostaining in dendritic processes of the melanocytes directs towards *MYO5A-NTRK3* fusion [29].

### 5.1. Spitz Neoplasms with NTRK1 Fusions

**Figure 4:**
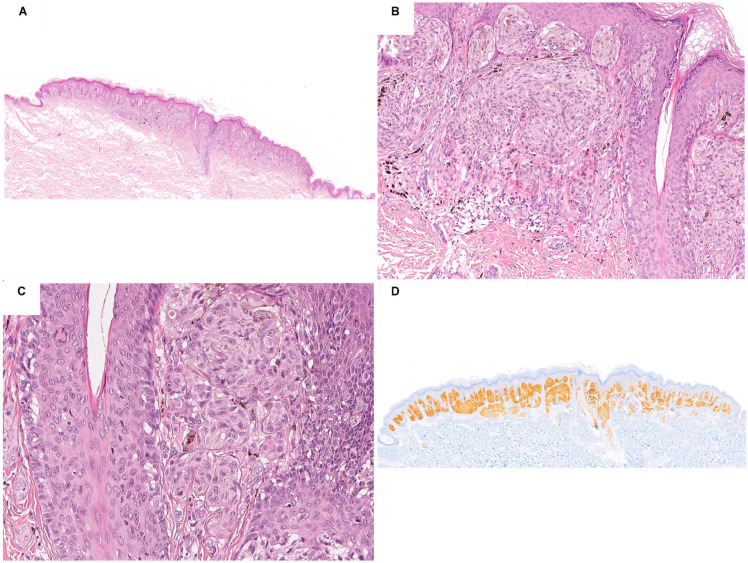
Photomicrographs of an atypical Spitz tumor harboring NTRK1 fusion showing a slightly raised, compound, and symmetric melanocytic proliferation with filigree-like rete ridges, flat-base silhouette, and lobulated nests of epithelioid and spindle melanocytes with moderate nuclear pleomorphism (**A**–**C**) H&E staining; original magnification 12.5×, 100×, and 200×, respectively). Pan-TRK (clone EPR17341) immunohistochemistry showing a diffuse cytoplasmic staining ((**D**) original magnification 12.5×).

Most Spitz neoplasms harboring *NTRK1* fusions are compound or dermal exophytic and symmetric lesions with thin and elongated rete ridges (filigree-like rete ridges), flat-base or wedge shape silhouette, lentiginous proliferation, lobulated nests, rosettes-like structures, and exaggerated maturation of epithelioid and/or spindle melanocytes with mild to moderate nuclear pleomorphism (Figure 4) [5,11,28,33]. Kamino bodies are frequently encountered, while mitoses are rare [5,11,13,28,33,76]. Like *ALK*-fused Spitz neoplasms, a plexiform pattern characterized by intersecting fascicles of spindle melanocytes can be observed in some cases [76].

### 5.2. Spitz Neoplasms with NTRK2 Fusions

The only reported *NTRK2*-fused SN belonged to histological variant of pigmented spindle cell nevus (aka nevus of Reed). The lesion is junctional, with hyperplastic epidermis, large nests with peripheral clefts composed of pigmented spindle melanocytes with abundant eosinophilic cytoplasm and elongated or oval nuclei [30]. Neither nuclear pleomorphism nor mitoses are observed [30]. Kamino bodies are widely distributed [30].

### 5.3. Spitz Neoplasms with NTRK3 Fusions

*NTRK3*-fused Spitz neoplasms are mostly compound or dermal, with epidermal hyperplasia and dome-shaped silhouette [29]. Depending on the underlying fusions, the lesions show different morphology. Indeed, cases with *ETV6-NTRK3* fusions are predominantly composed of epithelioid melanocytes with distinct cell borders, abundant eosinophilic cytoplasm, and pleomorphic nuclei, arranged in large coalescing and lobulated nests [29,33]. The constituent melanocytes of *MYO5A-NTRK3*-fused lesions, instead, are homogeneously spindled and organized in a fascicular to plexiform growth pattern [29]. Palisading resembling Verocay bodies and rosettes-like structures are occasionally seen [29]. Lesions with *MYH9*-*NTRK3* fusion are characterized by moderately large epithelioid melanocytes syncytially arranged with central desmoplastic stroma and peripheral collagen trapping [29].

The prognosis of Spitz neoplasms harboring *NTRK1* or *NTRK3* fusions is invariably favorable. Although rare cases of neoplastic cellular deposits in regional lymph nodes occur, no distant metastases and adverse outcomes have been reported so far [68,86].

## 6. Spitz Neoplasms with *RET* Fusions

*RET* proto-oncogene is located on chromosome 10q and encodes a tyrosine kinase receptor implicated in the PI3K-AKT, RAF-MEK1/2-ERK1/2, and PLCγ1 pathways [13,92]. *RET* fusions have been found in about 3–4% of Spitz neoplasms with the partner genes *CCDC6, KIF5B*, *LMNA*, *GOLGA5,* and *MYO5A* [13,31,85,93]. For their detection NGS or FISH analysis is required.

The few *RET*-fused Spitz neoplasms so far described are mainly compound symmetric lesions with epidermal hyperplasia, a plaque-like silhouette, large expansile nests of dyscohesive, intermediate-sized, and monotonous predominantly epithelioid melanocytes characterized by mild to moderate nuclear atypia [13,93].

Despite *RET* fusions have been found in SN, AST, and MST, the prognosis is favorable since the available follow up were uneventful [13,31,73,85,93].

## 7. Spitz Neoplasms with *MET* Fusions

*MET* proto-oncogene resides on chromosome 7q and encodes a tyrosine kinase receptor involved in the PI3K-AKT, RAF-MEK1/2-ERK1/2, PLCγ1, and β-catenin pathways [26,67]. Even though only a handful of *MET*-fused Spitz neoplasms have been reported up to date, the list of identified partner genes is quite long: *TRIM4, ZKSCAN1, LRRFIP1, PPFIBP1, EPS15,* and *DCTN1* [26,31,87]. In this setting, NGS and FISH are the available options for the recognition of the *MET* fusions.

The morphologic features of Spitz neoplasms harboring *MET* fusions are not specific. Indeed, most of the reported cases are compound or intradermal, symmetric, and dome-shaped lesions with epidermal hyperplasia, large nests of intermediate to large epithelioid or spindle melanocytes with pericellular clefting [26].

*MET* fusions have been found in SN, AST, and MST, but all cases with available follow up behaved indolently [26,31,87].

## 8. Spitz Neoplasms with *MAP2K1* Mutations

*MAP2K1* proto-oncogene is located on chromosome 15q and encodes MEK1, the serine-threonine and tyrosine kinase directly downstream of RAF, which in turn phosphorylates ERK in the RAF-MEK1/2-ERK1/2 pathway [34]. The molecular background of *MAP2K1* mutated lesions typically consists of in-frame deletions, that lead to an impaired MEK activation through RAF-dependent, RAF-regulated (conferring resistance to RAF inhibitors), or RAF-independent (insensitive to allosteric MEK inhibitors) possible mechanisms [37,94]. As for Spitz neoplasms, most *MAP2K1* mutations involve exons 2 and 3, especially as in-frame deletions (p.E102_I103del. and p.I103_K104del), removing an autoinhibitory domain of the protein thus leading to a constitutive activated state unresponsive to feedback inhibition by RAS and RAF (class II in-frame deletions) [36]. However, these alterations seem to be extremely rare in this context. To date, only few cases of *MAP2K1*-mutated Spitz neoplasms have been described: Victor et al. reported a single case, Kerckhoffs et al. 4 cases, Sunshine et al. 6 cases, Donati et al. 4 cases, and Kervarrec T et al. a single case [33,34,35,36,37]. Sanger or NGS analysis is needed for the detection of the mutations.

Clinically, Spitz neoplasms harboring *MAP2K1* mutations occur as small, flat or slightly elevated, pigmented lesions on the lower extremities of young patients, with a substantial female preponderance (M: F ratio 1: 2) [34,35,36,37]. Despite the attempts made to find relevant genotype-phenotype correlations within the framework of these lesions, this purpose has been greatly frustrated due to the very small sample size of the series, with the consequent wide variability of the observations [35,36]. Nevertheless, some morphologic features recur among the histological descriptions. Spitz neoplasms with *MAP2K1* mutations seem to be hallmarked by a tendency toward a compound or intradermal wedge shape silhouette, with plexiform architecture and with convergence around the adnexa and the neurovascular bundles of nests composed of large epithelioid cells with vesicular nuclei and moderate to severe nuclear pleomorphism [34,35,36]. Other histological findings comprise heavy pigmentation, stromal accumulation of melanin and melanophages, lack of epidermal hyperplasia, and poor maturation [36,37]. Of interest, Donati and co-workers also described the association between the cytological features of a Spitz neoplasm and the architecture of a dysplastic nevus, matching the description of the so-called SPARK nevus [37]. It has been suggested that this morphological heterogeneity of Spitz neoplasms harboring *MAP2K1* mutations resides in the effects of the secondary genetic hit on the phenotype [36]. Indeed, most cases harbor also other passenger mutations in well-known oncogenes and tumor-suppressor-genes, such as *BRAF, IDH1, BAP1,* and *NF1* [36].

*MAP2K1* mutations can be found in both benign and malignant Spitz lesions but are more common in AST and MST [34,36,37]. An overt malignant phenotype is mainly observed in cases with concurrent molecular aberrations involving *HRAS, CDKN2A, ARID1A,* or *NO*TCH2, or with copy number gains of 6p [35]. Nevertheless, no recurrences or adverse outcomes have been noted during the follow up of the patients [34,35,36,37].

## 9. Spitz Neoplasms with *MAP3K8* Fusions

*MAP3K8* proto-oncogene resides on chromosome 10p and encodes a serine-threonine and tyrosine kinase able to directly activate ERK1 and ERK2 in the RAF-MEK1/2-ERK1/2 pathway [95,96]. The kinase domain sequence of MAP3K8 is positioned between exons 1–8 of the gene, while the inhibitory C-terminal domain sequence is located in exon 9 [95,96]. This last exon is crucial because necessary for the proteasomal degradation of the enzyme [97]. Moreover, the C-terminus carries out its inhibitory activity by covering the kinase domain of MAP3K8 when is inactive, thus avoiding the phosphorylation of MEK1 and MEK2 and signal propagation [97,98]. The removal of the final exons of *MAP3K8* through truncation or fusion with other gene partners invariably results in an oncogenic protein product with intact kinase domain but lacking its inhibitory controls that fuels the RAF-MEK1/2-ERK1/2 pathway [73]. Several *MAP3K8* fusion partners have been reported so far among Spitz neoplasms: *CDC42EP3, CUBN, STX7, SVIL, DIP2C (83), UBL3 (83), SPECC1, ATP2A2, CCNY, ZFP36L1, GNG2, LINC00703, MIR3681HG, PCDH7, PIP4K2A, PRKACB, SFMBT2, SLC4A4,* and *SUBN* [5,31,32,34,73,99]. *MAP3K8* fusions can be investigated with RNA-based NGS or FISH.

Clinically, *MAP3K8*-fused Spitz neoplasms usually present as exophytic pigmented lesions on the lower extremities of patients in a wide age range, with a slightly predominance of females [32,99]. Most cases appear as compound asymmetric lesions with epidermal hyperplasia, dome-shaped or nodular silhouette, with a predominantly nested junctional component [5,31,32,33,34,73,99]. Cells are almost always epithelioid, characterized by abundant eosinophilic cytoplasm, enlarged nuclei with a uniformly dispersed chromatin, and prominent nucleoli [32,33,99]. Worrisome features, such as epidermal ulceration, full thickness Pagetoid spread of melanocytes in the epidermis, moderate to severe nuclear pleomorphism, lack of maturation of the dermal component, deep mitoses, and presence of many scattered giant multinucleated melanocytes, are frequently observed in *MAP3K8*-fused Spitz neoplasms [32,33,99]. Indeed, most lesions are classified as AST or MST [32,33,99].

From a molecular point of view, AST and MST with *MAP3K8* fusions regularly harbor additional genetic aberrations, mainly 9p21 deletion [5,31,32,33,34]. This can be easily showed by focal or diffuse homogeneous lack of immunostaining of the melanocytes for p16 [5,31,33,34]. Of note, Kervarrec et al. found that AST and MST with severe cellular atypia and p16 loss have a very high probability to be *MAP3K8*-fused [33].

Prognostically, Spitz neoplasms with *MAP3K8* fusions associated with other molecular alterations may behave aggressively, with local tumor recurrence, lymph node involvement, and even patient death, albeit exceptional [32,34,73].

## 10. Spitz Neoplasms with *BRAF* Fusions

*BRAF* proto-oncogene is located on chromosome 7q and encodes the upstream serine-threonine and tyrosine kinase of the RAF-MEK1/2-ERK1/2 pathway [100,101]. The gene comprises a conserved region for the N -terminal cysteine-rich domains for the binding of RAS proteins, one for the serine-threonine-rich domains, and another one for the kinase domain [100,101]. The former two regions also have a kinase auto-inhibitory function [100,101]. Typically, in *BRAF* fusions these domains are lost and their controlling activity impaired [102,103]. The kinase, however, regularly work leading to an increased phosphorylation of the downstream MEK1, MEK2, ERK1, and ERK2 [102,103]. Various fusion partners are known: *MAD1L1, MLANA, MYO5A, MZT1, AKAP9, AGK, CLIP2, SKAP2, SLC12A7, BAIAP2L1, CEP89, CUX1, DYNC1/2, LSM14A, NRF1, SOX6, TRIM24, ZKSCAN1,* and *EML4* [5,13,68,73,79,84,104,105,106]. RNA-based NGS or FISH analysis is required for the detection of *BRAF* fusions.

Clinically, *BRAF*-fused Spitz neoplasms usually occur as pink papules mainly on the lower extremities followed by the upper extremities [104]. The age range is wide, but most patients are young, even if slightly older than those with other subtypes of Spitz neoplasms [104]. There is a clear female predominance (M:F ratio 1:2) [104]. Histologically, these lesions are mostly compound or dermal, with epidermal hyperplasia, plaque-like, wedge shaped, or nodular silhouette, and are composed of intermediate to large epithelioid melanocytes with amphophilic cytoplasm, vesicular nuclei, and prominent nucleoli [5,11,33,68,79,84,104,106]. Nuclear pleomorphism is frequently marked [5,11,33,68,79,84,104,106]. Spitz neoplasms harboring *BRAF* fusions may show a typical pattern characterized by a superficial hyper-cellular dermal component with sheet-like architecture along with a deep hypo-cellular dermal component with prominent desmoplasia [11,13,33,104,106]. Moreover, nevoid feature has been observed more frequently in *BRAF*-fused Spitz neoplasms than in the other subtypes, excluded the *NTRK1*-fused one [33].

As for the *MAP3K8*-fused subtype of Spitz neoplasms, most cases with fusions involving *BRAF* are diagnosed as AST or MST, harbor additional genetic aberrations, especially 9p21 deletions, *TERT* promoter mutations, and 6p25 copy number gains, and may have a very aggressive behavior [5,11,13,31,33,68,73,79,84,86,87,104,105,106]. Indeed, distant metastases have been reported in patients with *BRAF*-fused MST [68,84,105].

## 11. Conclusions

The morpho-molecular landscape of Spitz neoplasms is becoming clearer, with the identification of specific subtypes with recurrent characteristics thus providing the basis for a more solid and precise tumor classification. Histological features may already guide the choice of the immunohistochemical and/or molecular investigations to be performed in AST and MST to identify the underlying driver genetic alterations. This allows to confirm the Spitz nature of the lesions and to highlight the need of further molecular analyses in cases with either *MAP2K1* mutations or *MAP3K8* and *BRAF* fusions since these subtypes is commonly associated with aggressive behavior. Future studies should provide long follow up data to substantiate this approach.

## Figures and Tables

**Table 1:** Clinical, histological, immunohistochemical, and molecular features of Spitz neoplasms.

	Spitz Nevus	Atypical Spitz Tumor	Malignant Spitz Tumor
Clinical Features			
Age	Mean and median age: 21 years (range 2–69 years)	Can occur at any age; more common in younger patients (<40 years)	Can occur at any age (often >40 years)
Location	Most commonly affects the extremities	Occurs on extremities, trunk	Occurs on extremities, trunk
Description	Pink or reddish plaque, papule, or nodule.	Plaque or nodule Color variegation	Enlarged Plaque or nodule Color variegation Asymmetry Evolving lesion
Histological Features			
Size	≤5 mm	5–10 mm	>5 mm (often >10 mm)
Silhouette	Symmetric	Symmetric or asymmetric	Often asymmetric
Circumscription	Sharp	Often poor	Poor
Ulceration	Absent	Possible	Often present
Epidermis	Hyperplastic	Often effaced	Often effaced
Nesting	Vertically oriented with clefting	Irregular	Irregular and confluent
Pagetoid spread	Sometimes central and focal	Sometimes diffuse	Extensive
Maturation	Present	Sometimes partial or absent	Absent
Necrosis	Absent	Usually absent	Sometimes present
Kamino bodies	Present	Often absent	Absent
Deep margin	Pushing	Mostly pushing	Often infiltrative
Inflammation	Inconspicuous	Patchy	Patchy or band-like
Cytological Features			
Shape	Enlarged epithelioid or spindle cells	Enlarged epithelioid or spindle cells with increasing atypia	Enlarged epithelioid or spindle cells with marked atypia
Pleomorphism	Absent or mild	Mild to severe	Moderate to severe
Cytoplasm	Ground glass	Granular	Granular
Nucleus	Finely dispersed chromatin	Heterogeneous chromatin	Hyperchromasia
Nucleolus	Distinct	Increasingly prominent	Large
Nuclear/cytoplasmic ratio	Low	Intermediate to high	High
Pigment	Superficial distribution	Variable distribution	Variable, often irregular distribution
Mitotic rate	0–2/mm^2^	2–6 mitoses/mm^2^	2–6 mitoses/mm^2^ (often > 6 mitoses/mm^2^)
Atypical mitoses	Absent	Mostly absent	Present
Immunohistochemical Features			
HMB45	Diminished with depth in dermal component	Diminished or variable with depth in dermal component	Deep staining common
Ki-67	<5%	5–15%	>15%
p16	Present (checkerboard pattern)	Sometimes diminished or absent	Often diminished or absent
Molecular Features			
CGH array	Isolated gains of 7p and 11q, tetraploidy	Often > 1 chromosomal abnormality Gains of 6p25	Often > 1 chromosomal abnormality Gains of 6p25
Loss of 9p21	Absent	Sometimes present (heterozygous or homozygous)	Often present (homosygous)
*TP53* mutations	Absent	Sometimes present	Often present
*TERT* promoter mutations	Absent	Sometimes present	Often present

**Table 2:** Morpho-molecular features of Spitz neoplasms and immunohistochemistry and/or molecular analyses useful for diagnostic confirmation. Histological features more characteristic of each molecular subtype are in bold.

Histological Features	Driver Alteration	Immunohistochemistry	Molecular Analyses
**Symmetric plaque-like lesion** Infiltrative borders Epithelioid and spindled large melanocytes Low grade cytological atypia Low mitotic rate **Desmoplastic stromal reaction** Predominantly intradermal growth	*HRAS* mutations 11p gains	HRAS^Q61R^ (clone SP174) not useful HRAS^WT^	NGS CGH or FISH
Symmetrical dome/wedge-shaped **large lesion** Epithelioid and spindle melanocytes Mild to moderate cytological atypia Low mitotic rate **Plexiform growth pattern** **Absent or scant pigmentation** **Absent or scant Kamino bodies**	*ALK* fusions	ALK (clones D5F3 and 5A4)	FISH or NGS
Plaque-like or nodular lesion Epithelioid and spindled melanocytes Mild to moderate cytological atypia Low mitotic rate **Prominent junctional component** **Transepidermal elimination of nests** **Adnexal involvement** **Numerous Kamino bodies**	*ROS1* fusions	ROS1 (clone D4D6)	FISH or NGS
**Lobulated nests** **Rosette-like structures** Epithelioid and spindled melanocytes Mild to moderate cytological atypia Low mitotic rate **Extreme maturation** **Filigree-like rete ridges** Predominantly junctional proliferation **Numerous Kamino bodies**	*NTRK*1 fusions	Pan-TRK (clone EPR17341)	NGS (FISH suggested if pan-TRK is positive but NGS is negative)
*Pattern ETV6-related:* **Large coalescing and lobulated nests** Epithelioid melanocytes Pleomorphic nuclei *Pattern MYO5A-related:* **Spindle melanocytes** **Fascicular to plexiform growth pattern** **Palisading and rosettes-like structures** *Pattern MYO5A-related:* Epithelioid melanocytes **Syncytial arrangement** **Central desmoplastic stroma** **Peripheral collagen trapping**	*NTRK3* fusions	Pan-TRK (clone EPR17341)	NGS (FISH suggested if pan-TRK is positive but NGS is negative)
Symmetrical, well-circumscribed proliferation with plaque-like silhouette Small to intermediate-sized epithelioid and Spindle melanocytes Low grade cytological atypia Nested growth	*RET* fusions	Not available	FISH or NGS
Symmetric dome-shape lesion Small to intermediate-sized epithelioid and spindle melanocytes Low grade cytological atypia Nested growth	*MET* fusions	Not available	FISH or NGS
**Penetrating nevus/dysplastic nevus-like architecture** Infiltrative margins **Large epithelioid cells with relatively high degree of cito-nuclear atypia** Poor maturation Lack of epidermal hyperplasia Stromal accumulation of melanophages **Plexiform growing pattern** **Hyperpigmentation** **Absent or scant Kamino bodies**	*MAP2K1* mutations	Not available	NGS
Dome-shaped or nodular lesion **Predominantly nested junctional component Ulceration** **Lack of maturation** Epithelioid melanocytes **Moderate to high grade cytological atypia** **High mitotic rate** **Giant multinucleated melanocytes**	*MAP3K8* fusions	Not available	FISH or NGS
**Superficial dermal sheet-like architecture** **Basal desmoplastic stromal reaction** **Lack of maturation** Epithelioid morphology **Moderate to high grade cytological atypia** **High mitotic rate**	*BRAF* fusions	Not available	FISH or NGS
